# Body condition among Svalbard Polar bears *Ursus maritimus* during a period of rapid loss of sea ice

**DOI:** 10.1038/s41598-025-33227-9

**Published:** 2026-01-29

**Authors:** Jon Aars, E. N. Ieno, M. Andersen, A. E. Derocher, Ø. Wiig, A. F. Zuur

**Affiliations:** 1 Norwegian Polar Institute, FRAM Centre, Tromsø, Norway; 2 Highland Statistics Limited, Newburgh, UK; 3 Department of Biological Sciences, University of Alberta, Edmonton, Canada; 4 Natural History Museum, University of Oslo, Oslo, Norway

**Keywords:** Climate change, Habitat loss, Body condition, Polar bear, Arctic, Climate sciences, Ecology, Ecology, Ocean sciences

## Abstract

Polar bears are only found in Arctic areas with sufficient access to sea ice and seals on which they prey. Studies have highlighted negative effects on condition and demographics in areas where sea ice cover is declining due to warmer climate, but condition of the Barents Sea polar bear population have not been examined yet. Loss of sea ice rate has been considerably higher here than in other areas with polar bears. We investigated variation in body condition index (BCI) among 770 adult bears, 1188 captures, in March-May 1995–2019, in Svalbard, Norway (western part of the Barents Sea). We assessed how intrinsic (female reproductive state, age) and both males and females, BCI declined until 2000, but increased afterwards, during a period with rapid loss of sea ice. In models including sea ice metrics and climate (Arctic Oscillation), there was no support for the predicted negative effect of warmer weather and habitat loss. This indicates a complex relationship between habitat, ecosystem structure, energy intake, and energy expenditure. Increases in some prey species, including harbour seals, reindeer, and walrus, may partly offset reduced access to seals. Our findings underline the importance not to extrapolate findings across populations.

**Supplementary Information:**

The online version contains supplementary material available at 10.1038/s41598-025-33227-9.

## Introduction

Climate change has impacted species and ecosystems worldwide, e.g. with a shift in species distribution^1,2^. Over the past decades, the increase in air temperature has been two to four times higher in the Arctic, depending on time span and season, compared to the global average^3–6^. This change has impacted Arctic ecosystems in diverse ways, with climate winners and climate losers, both in the terrestrial and the marine environment^7^. Loss of sea ice habitat in the high Arctic has been of particular concern, given the unique ecosystem and the sea ice dependent endemic species that are particularly vulnerable. Of mammals, those include several seal species, narwhal (*Monodon monoceros*), beluga (*Delphinapterus leucas)* bowhead whales (*Balaena mysticetus*), and polar bears (*Ursus maritimus*)^8,9^.

The Barents Sea (BS) area has experienced even greater temperature rises than other Arctic regions over the past few decades, with increases of up to around 2 °C per decade in some parts of the region^10^. This area, which is home to one of the 20 recognized polar bear populations (https://www.iucn-pbsg.org/), has also lost sea ice habitat at a rate of four days/year between 1979 and 2014, more than twice as fast as any of the other areas hosting polar bear populations^11,12^.

Sea ice loss has had major impacts on BS polar bear space use and diet, while significant negative effects on reproduction and survival are minor so far (see below). Bears captured in the Svalbard area (Norwegian part of the BS area) have two main space use strategies (ecotypes) with some bears being “local”, never leaving the Svalbard area. Other bears follow the sea ice, as it retreats in spring, and migrate hundreds of kms, often between the Russian archipelago Franz Josef Land and Svalbard, or north-east to the ice edge. Bears following that strategy have been termed “pelagic”^13^. The BS polar bears have been studied during an annual monitoring program including capture since 1987, with captures in the Svalbard area. The population was protected in 1973, and it increased significantly in size the following decades^14,15^. The total BS population was estimated at between 1900 and 3600 bears in 2004^15^, and may, based on a higher number of bears at the ice edge in 2015 than in 2004 (where the majority of bears are pelagic), have increased since then^16^. The number of local bears (about 240–260 individuals) seemed to be rather stable^16^. Negative effects on reproduction that may be explained by loss of sea ice habitat or correlated to climate are indicated for the oldest adult females^17^, but in general the population seems healthy^16^,www.mosj.no/en/indikator/fauna/marine-fauna/polar-bear.

The pelagic BS bears have difficulties reaching their maternity denning areas in east Svalbard in years of late sea ice formation (i.e., later than mid-November,^18^). These bears have a higher annual energy demand than local bears because they move more^19^, and also to a larger degree than in the past, have to swim long distances between the ice edge and the islands because they travel longer distances, as they follow the sea ice. They also have to swim longer distances between the ice edge and the islands because the sea ice is now on average 200–300 km further north than two-three decades ago^20^. Such long swimming trips are energetically demanding^21^. The pelagic bears may, however, hunt seals for longer periods of the year, unlike local bears, which move onto land when the ice breaks up until it reforms, a period that now lasts several months longer than it did a few decades ago^12^. Diet studies (based on stable isotopes and fatty acids) from Svalbard indicate that pelagic bears feed more on marine food sources than local bears, and that local bears feed more on terrestrial food now than earlier^22,23^. Observational data has shown a steep increase in number of bears spending more time on land in summer plundering bird nests in west Svalbard^24^, and satellite telemetry data has shown that adult females in east Svalbard spend less time hunting seals in front of glaciers in summer (due to lack of sea ice) and more time in areas with bird colonies^25^. Increased time on land in summer is consistent with findings from other polar bear studies (e.g.^24,26–29^). Recent work has modelled the link between energy stores and changing climate (variation in numbers of days bears feed) for polar bears, with focus on limits for survival and reproduction^30,31^.

In wild populations, body condition (BC) is a key determinant of individual fitness^32^.

Cerini et al.^33^ note that when a population starts to be negatively affected by changing conditions, changes in fitness traits (such as BC) are observed first, before changes in demographic rates (e.g., birth rates, survival), which may eventually lead to a decline in abundance. As such, monitoring changes in BC may give an early warning about negative demographic effects in a study population. Observed declines in BC together with negative effects on survival or reproduction over time as sea ice availability has decreased have been documented across several polar bear populations including Western Hudson Bay, Southern Beaufort Sea, and Baffin Bay^29,34–37^. In the Western Hudson Bay and Southern Beaufort Sea polar bear populations, loss of sea ice habitat has also been associated with declines in survival and reproduction^35,37,38^. In contrast, polar bears in the Chukchi Sea have not experienced a BC decline despite a shorter period with sea ice and an increased time where bears are on land^39^, and demographic rates have not declined^40^.

Polar bears have the ability to accumulate and store large fat reserves. They hunt seals intensively in spring and early summer, during which they can gain 70% of their annual energy intake in just a few months^41,42^. With a good fat reserve, even reproducing females giving birth and nursing cubs can be without food, for six months or more burning fat reserves^43^. Atkinson and Ramsay^44^ found that females on average reduced their body mass by 43% over 192 days during winter when reproducing. Derocher and Stirling^45^ found that females with body weight less than 189 kg in autumn did not produce cubs the following spring in western Hudson Bay.

BC in polar bears correlates with BC of their seal prey, both in areas where sea ice habitat loss has already impacted the population negatively, and in Chukchi Sea where it has not^39^. A study from Beaufort Sea indicates that, as bears spend more time on land, they shift from predating ringed seals *(Pusa hispida)* to a lower energy density diet, corresponding to periods of lower survival rates^46^.

In this study from the western Barents Sea, where (a) observations suggest that the number of bears has increased in recent decades^16^, (b) the local bears stay on land longer and are shifting to a more terrestrial diet which is less energy dense^24^, and, (c) the availability of sea ice has rapidly declined, resulting in a shorter spring and summer seal hunting season and an increased energy demand for pelagic bears due to greater distances between hunting, denning, and mating areas, we examine if BC has (1) declined over time, and (2) if annual variation in BC can be explained by variation in sea ice availability or climate, and (3) we explore how intrinsic variables like age, sex and reproductive state affect BC and the need to include those in the models to reveal the effects of the extrinsic variables and time. It is predicted that bears will invest in growth the early years of life, and females will have invested heavily in cubs during denning, while lone females may be able to allocate new resources into fat reserves.

## Materials and methods

### Capture, handling and measurements

**Fig. 1:**
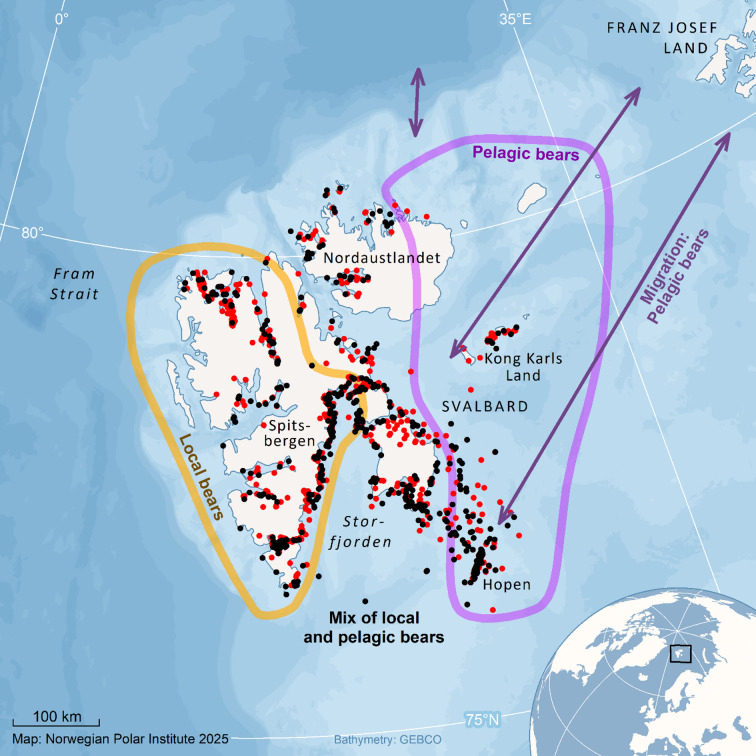
The map shows the area with captures (males = black dots, females = red dots). The yellow circle shows the area where bears captured usually belong to the “local” ecotype, the pink circle where bears belong to the “pelagic” ecotype. The area between is used by both ecotypes during the capture period in spring. Pink arrows indicate how pelagic bears migrate between Svalbard (where bears are captured) and the pack ice or Franz Josef Land. The background map was produced using ArcMap v10.8.2 (Esri, https://www.esri.com) with geospatial data from the Norwegian Polar Institute.

Polar bears were live captured in the Svalbard area, between latitude 74.3°N and 80.7°N, and longitude 11.5°W and 43.9°E. The captures took place in spring (between 22 March and 8 May), as part of the annual monitoring program of polar bears conducted by Norwegian Polar Institute in the area, from 1992 to 2019. The bears are part of the Barents Sea (BS) population. Pelagic bears captured in Svalbard commonly migrate to the Russian Franz Josef Land archipelago (Russia), or follows the marginal ice zone as it retreats north in summer and autumn. Pelagic females use land areas in Franz Josef Land or Svalbard for maternity denning^13,15^ (Fig. 1). Bears were live captured by immobilization using the drug Zoletil Forte following standard procedures^47^. A vestigial premolar tooth was extracted at first capture for age estimation^48^ except for bears initially captured as juveniles of known age. The data set initially included 1300 captures of adults (5 to 28 years old), with no more than one capture per individual each year. The animal handling protocols were approved by the Norwegian Animal Research Authority (FOTS) (permits ref: FOTS ID 31180) and the Governor of Svalbard (permits ref: 17/00389 − 13), and all research carried out according to guidelines and regulations. All methods are reported according to the ARRIVE guideline that are relevant for observational field studies including animal handling, and analyses of such data.

Morphometric measurements were taken from each bear during handling, including: (a) Body Length (BL, in cm): the dorsal straight line distance between the tail end (last vertebrae) and the tip of the nose, and (b) Axillary Girth (G, in cm) measured as the circumference at the axilla around the chest, with a rope tightened with a tension of approximately 0.5 kg. Body mass was estimated for each bear based on BL and G, based on correlations between measured weight and these parameters^49^, where weight (W, in kg) = 0.00003377*G^1.7515^*BL^1.3678^.

Since we did not have the measured body weight for females in the early years and only a few males, body mass was estimated for each bear based on BL and G. Using correlations between measured weight and weight predicted from BL and G was earlier estimated with an SE of 20 kg for larger males, or less (for smaller males, and females), among polar bears captured in Svalbard between 1990 and 2000^49^. Part of the variance around the predicted weight could be due to the fact that G and BL would not change immediately after a large meal, and since only a fraction of that meal is converted into the bear’s fat layer, it is debatable whether measured body weight or estimated body weight best reflects the BC of a bear. We used a body condition index (BCI) to estimate the fat reserves using estimated weight and size (BL) for polar bears from Cattet et al.^50^:

$${\mathrm{BCI}}=({\text{ln W}}--{\mathrm{3}}.0{\mathrm{7}}*{\text{ln BL}}+{\mathrm{1}}0.{\mathrm{76}})/(0.{\mathrm{17}}+0.00{\mathrm{9}}*{\text{ln BL}})$$

We also calculated the Quetelet’s index (W/BL^2^) following Rode et al.^36^, considered as an alternative to BCI.

### Sex and reproductive classes

Data from adult males and adult females were analysed separately. For adult females, we used a covariate representing reproductive state with three states. They were classified as being with cubs-of-the-year (Fc), with one-year-olds (Fy), or as alone or with two-year-olds (that would leave the mother shortly after capture, being weaned in spring) (F).

### Capture locations and date

Capture locations (Location) were recorded as decimal degrees. Although it was a goal to cover a wide and representative area of the archipelago, both sea ice conditions and weather dictated where bears could be captured, in addition to logistics (reach of helicopter from settlements or vessel). The number of pelagic bears would be higher east and south-east while bears in central areas would have a mix of local and pelagic bears (Fig. 1).

Date of capture (Day) represents the time in spring. Since capture never stared before March, and to avoid the issue with leap years, this variable was set such that Day 1 = 1 March, Day = 2 was 2 march and so on.

### Climatic and sea ice data

The AO is a large-scale climate index reflecting pressure and temperature north of 20°N^51^. It influences wind regimes in the Arctic, and thereby sea ice drifts. When AO is high, more sea ice drifts out from the Arctic basin through the Fram strait, and when AO is low, more sea ice tends to be trapped in the Arctic basin^52^. Satellite-derived sea ice metrics like extent do not necessarily correlate well with AO values, possibly because AO impacts other sea ice features as thickness, ice rafting, and presence of open leads more, as discussed in Nacari et al.^17^. These features may also be important both for the bears and their prey (sea ice associated seal species), and for the bears ability to catch the seals. Based on earlier work with the same population^14,17^, we therefore used the AO indexes for the climate variables: winter AO = October before capture to January of the year of capture (average of the monthly values), spring AO = April to June of the year of capture (average of the monthly values). As conditions in spring could be affected by conditions of the previous spring, when bears accumulate most of their fat reserves^53^, we included the AO spring index in the year before the capture year, year t-1 (SpringAOLag1).

For sea ice available variables, we used the same data and approach as Naciri et al.^17^, where sea ice extent in an area surrounding Svalbard based on a 95% minimum convex polygon of 135 different adult female bears (data from satellite telemetry collars) between 1989 and 2021 was used. Using sea ice extent rather than concentration is consistent with other studies on polar bears^31,54^. Cells of 12.5 km^2^ or 25 km^2^ (depending on year) were scored a 1 if sea ice cover was > 30%, and 0 if not, to calculate the cover in the designated area daily (see^17^. A transition date for sea ice extent was defined as halfway between the 30-day minimum (September) and maximum (March) extent over 1990–1999. For each year, BreakUp was defined as the date when sea ice extent had been below the transition date for 5 consecutive days, and likewise, FreezeUp as the date when sea ice extent had been above the transition date for 5 consecutive days. BreakUp dictates how long the bears can hunt on the sea ice in late spring or summer, FreezeUp when bears that have been forced on land can again enter the sea ice. Consistent with the exploration of AO effects (see explanation above, and Table 1), we explored the possible effects BreakUp in the year before capture (BreakUpLag1). The length of the ice-free season (IceFreeDays, the period bears may be forced to remain on land) was defined at the period between BreakUp and FreezeUp : To limit the number of tests and due to the correlation between the sea ice variables, FreezeUp was used to calculate IceFreeDays, but excluded from the analyses as a separate variable. The IceFreeDays represented the year preceding capture, a period during which bears stranded on land may be without access to seals and must rely on their fat reserves or compensate by exploiting terrestrial food resources.

### Statistical analyses

The core of our analysis involved the application the following model:

$$\begin{aligned} & {\mathrm{BC}}{{\mathrm{I}}_{ij}}=N({\mu _{ij}},{\sigma ^2}) \\ & {\mu _{ij}}={\mathrm{Intercept}}+{\mathrm{Covariete}}{{\mathrm{s}}_{ij}}+{\mathrm{Dependenc}}{{\mathrm{y}}_{ij}} \\ \end{aligned}$$

Where BCI_*ij*_ represents the j^*th*^ observation of the BCI for animal *i*, and N() stands for a normal distribution with mean *µ* where *µ* is modelled as a function of covariates and dependency.

We identified a large number of potential covariates, and data exploration indicated a certain degree of collinearity. We formulated 19 competing underlying biological hypotheses that explained BCI in terms of covariates (see Table 1). The Covariates_*ij*_ term in the model was quantified accordingly. Data exploration also indicated potential non-linear covariate effects, for which we applied smoothing functions. Consequently, our model is a generalised additive model (GAM) with a normal distribution and identity link function^55^. The Gaussian likelihood is standard for modelling condition indices where residual variation is assumed to be symmetrically distributed around the fitted mean. Moreover, model validation diagnostics for the Gaussian GAM with spatial correlation (residual checks, posterior predictive assessments, and spatial residual patterns) confirmed that this specification provided an adequate fit.

Recognizing the possibility of capturing the same bear multiple times across different years, we treated the individual bear as a random effect in our models. This approach allowed us to account for the within-subject correlation, acknowledging that repeated measurements from the same bear are more likely to be similar than measurements from different bears. Consequently, in all models, the ‘Dependency’ part contains a random intercept. This random intercept is assumed to be normal distributed with mean 0 and variance σ^2^_Bear_. Some of the models (4 and 8–10, Table 1) included a spatial component in the ‘Dependency’ term. These spatial models allowed us to explore and quantify any spatial patterns or dependencies in the bear captures, providing a deeper understanding of the spatial dynamics. It should be noted that the spatial dependency term may also represent the effect of covariates not included in the model.

For the implementation of our models, we employed the “Integrated Nested Laplace Approximation (INLA)” method using the R-INLA package (www.r-inla.org). R-INLA provides a computationally efficient alternative to traditional Markov Chain Monte Carlo (MCMC) methods for Bayesian inference, which is particularly beneficial for models with spatial dependency.

In R-INLA, the integration of smoothers, random effects, and spatial correlation components is facilitated by the careful selection of priors. These priors regulate the influence that each term exerts on the model, essentially controlling the amount of information that is attributed to specific model components. By carefully choosing priors, we can finely tune the balance between model flexibility and the risk of overfitting, ensuring that each term contributes appropriately to the model’s overall predictive performance. For smoothers, we implemented random walk models of order 2 (RW2). The priors for these smoothers were specified according to the penalized complexity (PC) framework^56^. We adopted the recommended values from the RW2 documentation in R-INLA, ensuring an optimal balance between model flexibility and overfitting.

For the random effect associated with bear identity, σ_Bear_, we also employed a penalized complexity prior. This choice was made to effectively capture the variability among individuals while avoiding undue model complexity. Further details on the prior specification for σ_Bear_ are available in the Supplementary Information.

Incorporating a spatial correlation component into our model necessitated the construction of a spatial mesh, a critical step in defining the spatial structure within R-INLA. The development of this mesh involved careful decisions regarding mesh density, which directly influences the granularity of spatial analysis, and the selection of appropriate priors for the spatial parameters.

Literature^57^ suggests that the outcomes derived from R-INLA are sensitive to these choices, particularly the mesh resolution and the specifications of priors for the spatial parameters. To ensure the robustness of our results against these methodological variables, we conducted sensitivity analyses. These analyses entailed running our model with varying mesh sizes and different prior values for the spatial parameters. The consistency in results across these varied settings provided confidence in the stability and reliability of our findings, indicating that our conclusions were not unduly influenced by the specific choices made in mesh construction and prior selection.

The spatially structured variation in BCI, included in several models (4 and 8–10; Table 1), is shown in Fig. 4. In R-INLA, this structure is implemented as a *spatial random field* (SRF), represented in our study by the variable *Location*. The mesh defines the spatial framework used to estimate the correlation associated with *Location*, enabling the model to capture spatial dependence in the data. In the models (fitted separately for males and females), *Location* represents spatial structure not explained by the measured covariates, reflecting large-scale spatial patterns in body condition across Svalbard. The spatial models thus have the form:

$${\mathrm{BCI}}={\text{covariate effects}}+{\mathrm{Location}}+{\mathrm{error}}.$$

Model selection was guided by both statistical and ecological considerations, ensuring that each model was both statistically robust and ecologically meaningful. We utilized two model comparison metrics to compare the fit of different models: Deviance Information Criterion (DIC) and the Watanabe-Akaike Information Criterion (WAIC), both appropriate for Bayesian hierarchical models such as those fitted with R-INLA. Additionally, we conducted diagnostic checks and validation procedures to assess the adequacy and predictive performance of our models. The Information Theoretic Approach was conducted to select the best candidate models^58^. We utilised the DHARMa package^59^ and checked the scaled quantile residuals for homogeneity by plotting them against each covariate, and we verified the absence of any remaining spatial dependency.

**Table 1:** A priori candidate models with explanatory variables explaining body condition (BC) of adult male Polar bears (A), and adult female Polar bears (B). Individual bears were added as a random variable to each model (for simplicity not expressed below).

A. Males		
Model	Expression	Hypothesis
M1	Intercept only	Variation in BC is not explained by any variables
M2a	WinterAO + SpringAO	BC is impacted by climate in winter preceding capture and spring of the capture year
M2b	SpringAOLag1 + WinterAO	Climate in spring, when bears accumulate fat reserves, and in winter, is important for BC the following year
M3a	BreakUp	Sea ice break up affects hunting success of that year
M3b	BreakUpLag1	Sea ice break up affects fat reserves the following year
M3c	IceFreeDays	Duration of the season with no sea ice impact opportunities to hunt seals the following year
M4	Location	Bears in different locations have different hunting success rates and energy demands
M5	Year	There is a time trend due to change in factors not explained by other available variables
M6	Day	BC changes with time in spring, due to changes in activity and prey availability
M7a	WinterAO + SpringAO + BreakUp	Climate and sea ice availability effects are additive
M7b	WinterAO + SpringAO + BreakUpLag1	As 7a, but fat accumulation the previous year is more important than the capture year
M7c	WinterAO + SpringAO + IceFreeDays	As 7b, but days forced on land better predict condition than date of sea ice break up
M8a	WinterAO + SpringAO + BreakUp + Day + Location	Climate, sea ice, time in spring, and bear location all impact BC
M8b	WinterAO + SpringAO + BreakUpLag1 + Day + Location	As 8a, but fat accumulation the previous year is more important than in capture year
M8c	WinterAO + SpringAO + IceFreeDays + Day + Location	As 8b, but days forced on land are a better predictor of BC than time of sea ice break up
M9a	WinterAO + SpringAO + BreakUp + Day + Location + Age	As 8a, but in addition, bears of prime age likely do better than other bears
M9b	WinterAO + SpringAO + BreakUpLag1 + Day + Location + Age	As 8b, but in addition, bears of prime age likely do better than other bears
M9c	WinterAO + SpringAO + IceFreeDays + Day + Location + Age	As 8c, but in addition, bears of prime age likely do better than other bears
M10	Year + Age + Day + Location	A time trend explains variation better than external factors of climate and sea ice

B. Females		
Same as for males, with addition of reproductive stage (WithCubs, Fc = with cubs, Fy = with yearlings, F = lone females or with two-year olds) to all models. Corresponding models will be termed with F for females replacing M (for males), e.g. the corresponding model of M1 for males with intercept only will for females be F1 with intercept + WithCubs.		

## Results

### Variation in sea ice habitat and climate

On average, sea ice BreakUp happened about a month earlier after 2005, compared to 1995–2000, and the number of IceFreeDays increased by several months (approximately 100 days), mostly due to formation of ice late in fall or into next winter (Supplementary Fig. 1). Up to 2006, no years had > 175 IceFreeDays. After 2006, five years had > 200 IceFreeDays. Given the earlier sea ice break up in years when spring AO > 0.4, the relationship between AO and date of sea ice BreakUp supported the theory that more sea ice can leave the Arctic through the Fram Strait (see above) when AO values are high (Supplementary Fig. 2).

### Initial explanatory analyses of capture data

The data (1300 captures) was plotted for visual inspection. Due to few captures, and in a restricted local area of Svalbard, we omitted data from 1992 to 1994 (*n* = 62 captures) from further analyses. Further, we excluded captures west of 0° and east of 35°E longitude (another 50 captures), to ensure a relatively homogenous data set regarding area covered over time. This left us with 1188 captures of adult males and adult females. Plotting the two possible response variables (BCI and Quetelet’s) revealed a very tight relationship, which gave no reason to believe that one would provide a significantly different result than the other. BCI has been the more commonly used index in studies of polar bears earlier^36,50^. We thus decided to use BCI to represent bear condition (BC) in all following analyses. Data exploration indicated the absence of outliers in all variables. Regarding collinearity, we observed minor associations between some variables, with correlation coefficients reaching up to 0.6. However, the VIF values remained < 3, suggesting manageable multicollinearity. Temporal dynamics were evident in some covariates, such as the springAO, and demonstrated a clear temporal pattern, as did the response variable BCI. The spatial sampling locations varied across the study period. The number of annual captures (in 1995–2019) varied from 5 to 66 for males, and from 13 to 47 for females, indicating that the dataset was not sufficiently robust for a full spatial-temporal modelling. Therefore, we modelled space and time separately: spatial dependence was captured through the spatial random field (based on capture *Location*), while interannual variation was represented by including *Year* as a covariate. The results from the analyses conducted in INLA are reported separately for males and females below. Model validations indicated that the optimal models complied with all assumptions.

**Fig. 2:**
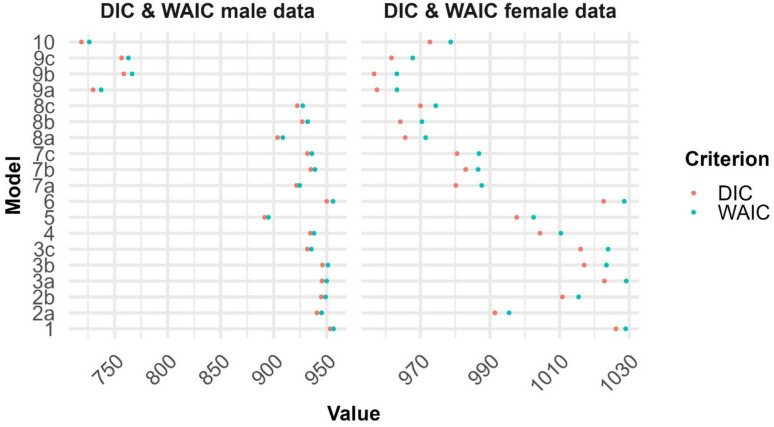
The figure is showing the DIC and WAIC values for the competing models for males (left) and females (right).

### Body condition of adult males

The male data consisted of 524 captures of 330 individuals, with maximum eight captures for individual bears. The DIC and WAIC values for all alternative models are presented in Fig. 2 (left panel). There are four competing male models that clearly stand out with lowest values (i.e., M10, M9a, M9c and M9b). We focus on the results of model M10 that had the lowest DIC and WAIC values, and model M9a that had considerably lower DIC and WAIC values than the alternative M9b and M9c. The two latter models are presented in Supplementary Fig. 3a, Supplementary Fig. 4a, and briefly discussed below.

**Fig. 3:**
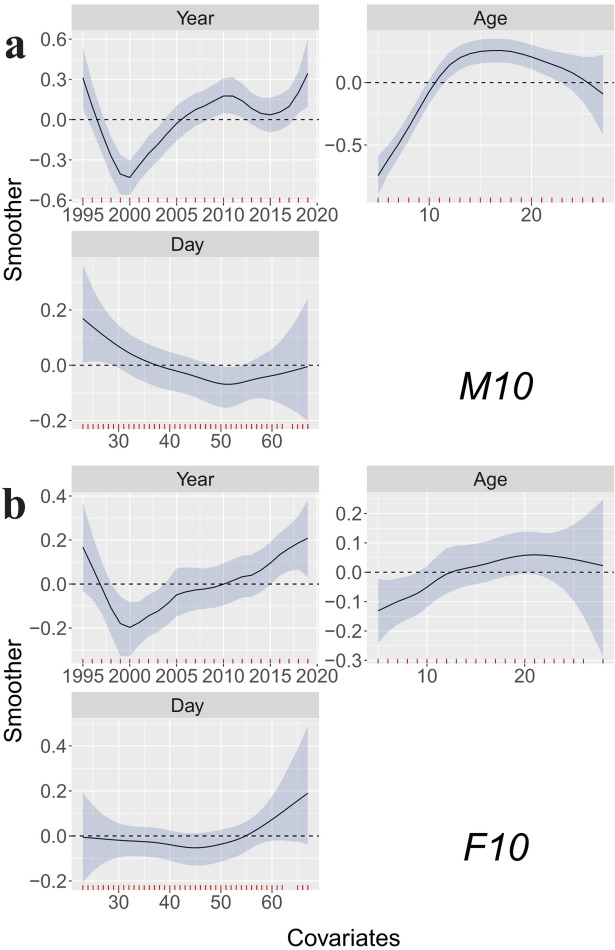
Smoothers for the partial effects for (**a**) model M10 (males) upper panel, and for (**b**) model F10 (females) lower panel, including year of capture (Year), age at capture (Age), and day of capture (Day, where 1 March = 1). The small red ticks above the x-axis denotes data available.

**Fig. 4:**
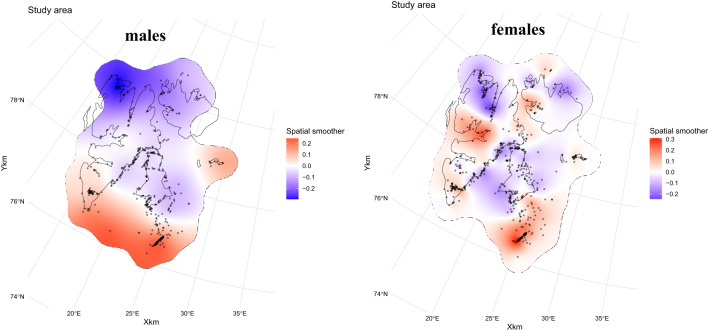
Spatial random fields (SRFs, variable Location) from the optimal INLA models for males (left, Model M10, M9a-c, M8a-c, M4) and females (right, Model F10, F9a-c, F8a-c, F4), showing the estimated spatial component of body condition index (BCI) across the Svalbard area. The SRF (Location) represents spatially structured variation in BCI that is not explained by the covariates (i.e. Year, Age, and Day in M10 and F10), and was estimated jointly with the covariate effects in a Gaussian GAM with spatial correlation. Positive values (red areas) indicate regions where bears were in better condition than predicted by the fixed effects, and negative values (blue areas) indicate areas of lower condition. Black dots show capture locations. Contour lines of Svalbard were generated using the **rnaturalearth** package^60^ in R.

**Fig. 5:**
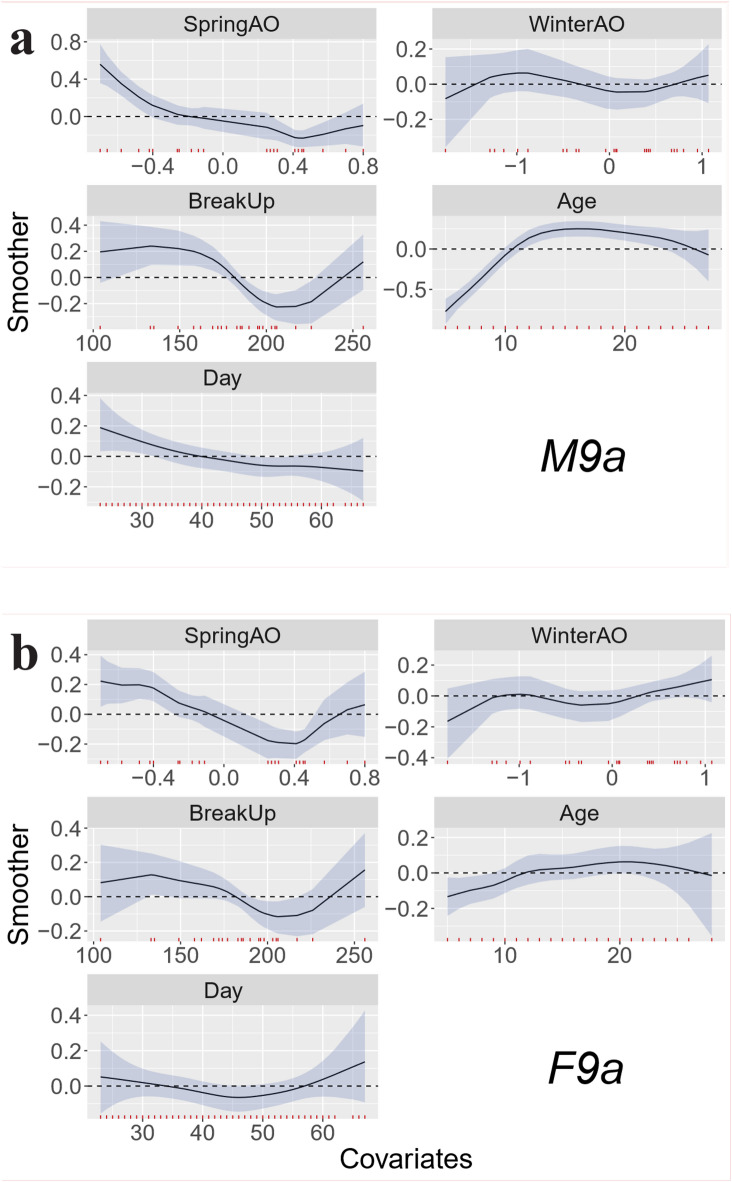
Smoothers for the partial effects for the explanatory variables (covariates) for (**a**) model M9a (males, upper panel), and (**b**) model F9a (females, lower panel), including index of Arctic Oscillation in spring (SpringAO), in winter before capture (WinterAO), time (Julian day) of sea ice break up (BreakUp), age at capture (Age), and day of capture (Day, where 1 March = 1). The small red ticks above the x-axis denotes data available.

Model M10 (Fig. 3a), considering age and time in spring, the smoothing function Year decreases sharply from 1995 to about 2000, then increases steadily over the next decade, reaching a value close to the initial value, before showing only modest variability in subsequent years. The age effect shows a sharp increase until males are 12 years old, and varies little after that, but with a tendency of a decline with old age (e.g., > 20 years, but with small sample size). Through the season, there is a decline in the effect on BCI from mid-March until after mid-April (1 April = day 32), then a possible slight increase towards the end of April/start of May, but with high uncertainty. The model predicts males to be in better condition in the south and east Svalbard compared to in northwest (Fig. 4, left panel). For model values, see Supplementary Table 1.

ln model M9a (Fig. 5a), the best alternative model to M10, year was replaced by climate and sea ice indices. SpringAO shows a generally decreasing association with BCI across most of its range, with a modest rebound at its higher values. In contrast, WinterAO exhibits a weak, non-monotonic pattern with wide uncertainty intervals that largely include zero; any apparent upturns at the extremes occur where data are sparse. In most years, sea ice BreakUp happened between Julian day 150 and 200 (30 May and 19 July), and the modelled BCI markedly declined with later BreakUp, in contradiction with expectation. The relationship between BCI and both Age and Day (time in spring) did not change form from model M10. For model M9a, values are provided in Supplementary Table 2. In model M9b (Supplementary Fig. 3a), examining the relationship between BCI and time of BreakUp the year before capture (BreakUpLag1), instead of the year of capture, showed the same relationship where BCI decline with later sea ice BreakUpLag1. In accordance with that finding, longer ice-free periods in the year before capture (IceFreeDays) also corresponded to higher BCI-values (model M9c, Supplementary Fig. 4a).

### Body condition of adult females

The female data consisted of 664 captures of 440 individuals, with individual bears being captured between one and eight times. The two best models were F9b, F9a, with almost identical DIC and WAIC values (Fig. 2). We focus on F9a as it is comparable to M9a, the best of the male models that incorporated climatic and sea ice indexes. Similarly, we briefly discuss model F9b and F9c. Again, to be able to compare male and female data, we also focus on model F10, the best of the models that did not include sea ice and climate variables. The female models include reproductive state as a fixed effect. The partial effects from the model are as we presumed: Fc has a lower BCI (-1.23, set as intercept) than Fy (BCI = 0.15), that again has a lower BCI than F (BCI = 0.35), see Supplementary Table 3. The results of model F9a (Fig. 5b) are similar to those of male data (M9a, Fig. 5a): when other factors are accounted, the youngest bears have the lowest BCI, although the increase is less marked in females and reaches its asymptote around 10 years of age. As for males, there is a tendency for BCI to decline with the smoothed function Day (partial effect) until after mid-April beyond which, with limited data and high uncertainty, BCI possibly increases in late April to early May. The partial effect of Spring AO shows the same relationship as for males, with declining BCI with increasing AO except for the few highest AO values (> 0.4), where BCI increases again. Furthermore, accounting for other variables, for most of the range, BCI values decreases with later dates for sea ice break up in spring and summer in the year of capture. For model F9a, values are provided in Supplementary Table 3. The models F9b and F9c show results consistent with those obtained for males, where, when other covariates are adjusted for, BCI declines with timing of sea ice break up the year prior to capture and increases with longer periods without sea ice in the year prior to capture (Supplementary Fig. 3b, Supplementary Fig. 4b). Model F10 has the same pattern for the effect of female age and day in spring as in model F9a. For year, accounting for any covariates, the female model follows the same marked decrease in BCI in the early years (1995 up to around year 2000) as for males, and a steady increase after that. The model values for F10 are provided in Supplementary Table 4. The spatial variability shows a partly consistent pattern with males indicating that bears were in lower condition in northwest Svalbard, and with the highest BCI in southeast. However, the spatial smoothing modelled for females also shows higher BCI values along the west coast of Spitsbergen (west Svalbard), about halfway up the island. The spatial pattern is patchier and more complex than for males.

## Discussion

Sea ice loss has been greater in the Barents Sea area during the period covered by this study than anywhere else where polar bears live^11,12^, and deterioration in body condition should be the first sign of change before the negative demographic effects, which have been observed in several other polar bear populations across the Arctic^61,62^. Considering these factors, we predicted that body condition in adults would decline over time and that bears would be leaner in years with less available sea ice or in the spring following such years. A reduction of more than two months in duration of the annual sea ice season between 1995 and 2019 in the Barents Sea area led to significant ecological changes for the polar bears (e.g., loss of denning areas:^18^, significant northward shifts in sea ice habitat:^20^, and genetic effects explained by restricted gene flow:^63^). However, sea ice loss did not lead to a reduction in BC among adult BS polar bears. Rather, after around 2000, following an initial negative trend from 1995, both males, and females of different reproductive categories, increased in body condition for the following two decades.

While the far best model for males did not include any sea ice or climate variable, for females, a more complicated model including both Arctic Oscillation (AO) in winter and spring, and the day of sea ice break up the year before capture, gave the best fit to the data. Contrary to our prediction, females were in poorer condition in the spring following a particularly late sea ice break up (after day 200 / 20 July). In the same model, the reduction in BC when spring AO ranges from − 0.4 to 0.4, could fit with the theory that more sea ice is trapped in the Arctic Basin when AO is low, and more ice leaves the area through the Fram Strait when AO is high. However, for spring AO values higher than 0.4, the BCI increased. Also, winter AO showed a positive relationship with BCI. Furthermore, we would expect that sea ice metrics in the year of sampling would be a better predictor than AO, if the AO indices operated as proxies for sea ice availability. Naciri et al.^17^ discussed the possible explanation that AO represented not only sea ice distribution, but also variability in sea ice thickness and structure (e.g. presence of open leads, ice movement) that could also be important for the bears (e.g., affecting energy use, hunting opportunities).

We did not provide a clearer understanding of the relationship between climatic indexes and sea ice habitat, and failed to find evidence for how habitat loss may negatively affect the condition of polar bears. Thus, our findings contrast with reports from other populations where loss of sea ice has had clear negative effects on polar bear condition, growth, or demography, in particular from Western Hudson Bay and the Southern Beaufort Sea (e.g.^35–38,64,65^), as well as current and predicted effects of polar bears in the Baffin Bay area^29^. However, our findings fit in with results from the Chukchi Sea area where bears are still in good condition despite less sea ice, explained by a very high biomass productivity including high densities of seals, a large continental shelf area, and the duration sea ice over the shelf^39^.

For males, the best models included a spatial component, with increasing conditions for bears captured further south and further east in Svalbard, and the lowest condition in the northwest. The best models for the females also included a spatial structure, and mostly consistent with the pattern seen for males. Pelagic bears are more likely to be captured in the southern and eastern areas of Svalbard. Biases in capture also favoured sampling of pelagic bears in the earlier years of the monitoring program (until just after 2000), both because researchers operated out of Hopen Island in southeast Svalbard and because sea ice still connected the ice edge and Svalbard almost year-round, allowing easy migration between sea ice hunting areas and the islands. In years with little connection between the ice edge and Svalbard, most bears encountered in Svalbard would be “local bears”^16^. The spatial gradient observed could thus be explained a) with pelagic bears being in better condition than local bears, and/or b) worse conditions for the local bears using the areas in NW Svalbard than further east and south. The increased condition over time, as the proportion of pelagic bears that were captured declined, favour the latter explanation. The area in NW has experienced a significant loss of sea ice in front of glaciers in later years, areas where bears can hunt ringed seals in spring and summer if sea ice is present^25,66,67^. Bears from this area have, in later years, frequently started to migrate down the west coast in early or mid-summer, returning north in late summer or fall (unpublished data). However, polar bears further south along the west coast of Spitsbergen (west Svalbard) still appear to be in good condition, despite these areas experiencing greater sea ice loss, warm water transported by the Gulf Stream, and water temperature rising by several degrees in recent years^68^.

Male condition depended heavily on age with a steep increase up until about 12 years, and a tendency for a decrease for older males after about 20 years of age. Males invest in growth several years beyond the age of adulthood^49^. A trade-off between investment and growth is thus a likely explanation, although younger individuals may be leaner also due to lower hunting success. In contrast, females in Svalbard have already invested most of their growth by the time they reach adulthood i.e., five years of age^49^. Consequently, the change in body condition was less pronounced than in males (models F9a and F10), with the youngest bears still being leaner.

Among adult males, body condition decreased from late March until a few days after mid-April, then increased through the remaining period for which data was available (until early May). Day (date in spring) was not included in the best model for females. A trend (with high uncertainty) with a decline through the first part of April was indicated in the alternative (F10) model, and later an increase in BC (as for males). The winter is a period with low activity and low intake of food for polar bears^69^, thus even bears not in maternity dens will usually lose weight over winter^69^. The main diet of polar bears in spring in Svalbard is ringed seal^70^, where the ringed seals give birth around early April. Their pups weigh on average just below five kg at birth, thus being a small meal for a bear. After about a month later, they exceed 20 kg^71^ thus being of a much higher energetic value. Older ringed seals may be more easily available later in spring, as they use more time on the ice when days get longer and air temperature higher^72^, although during the period for which bears in our study were captured, pup ringed seals were still by far the dominant prey^70^. The increasing availability of pup ringed seals of more significant energetic value thus likely explains the change in condition through spring to a large extent^53^. Another factor may be time allocated to hunting vs. mating activities. March through April is the main mating season, with a peak around 9 April for Svalbard^73^. More males are available for mating than females, as the females with cubs and yearlings do not mate. Fierce competition between the males^73^ may also be costly. Males may spend time searching for available females either over larger areas^69^, or more locally through tortious movements^74^ while females prioritize resource acquisition, a pattern typical among mammals^75^. The change in condition over time through spring shows the importance of standardized dates for field programs.

The polar bears in Svalbard have retained good body condition despite a significant loss of sea ice habitat after 1995. One possible explanation is that the density of ringed seals is higher in years with lower areas of sea ice, so that bears would use less effort for each seal taken (see e.g.^76^). Thus, even if the period when bears can hunt on the sea ice gets shorter, bears may be more efficient during that period. Polar bears have a unique ability to put on most of the fat reserves needed to survive and reproduce during a period of only a few months each year^69^. Furthermore, models that have attempted to predict how much time per year a polar bear can remain on land and survive have assumed that bears fast when stranded or that the use of terrestrial food sources is insignificant (^30,31,64^). Although this seems to be true for much of the Arctic (e.g.^42,77^), both the availability of different food sources on land and the competition with other predators vary with area.

In Svalbard, polar bears have been shown to prey more on bearded seals (*Erignathus barbatus*) in summer^70,78^. They use less time hunting ringed seals in front of glaciers, due to the loss of sea ice habitat there^25^, bearded seals can be hunted in summer also when sea ice is absent. In many cases, stranded whale carcasses may provide food for bears for over a year, and such carcasses are common in Svalbard^79^. Local bears in Svalbard have, in later years, increasingly eaten eggs and birds of common eider ducks (*Somateria mollissima*) and geese^24^. Svalbard reindeer (*Rangifer tarandus platyrhynchus*) have increased in abundance^80^, and in later years there have been many observations showing that polar bears are able to successfully hunt reindeer (e.g.^81^). Another food source that is now increasingly available is the walrus (*Odobenus rosmarus*) which now has recovered from earlier over-exploration^82^. Even though polar bears are rarely seen to successfully hunt walrus^69^, available carcasses may provide a good source of food. Along the west coast of Svalbard, early explorers noted that walruses were plentiful everywhere, and that polar bears were found in large numbers^83^. These areas have had a significant increase in number of bears in later years^24^. It is also interesting that females on the west coast, where the sea ice season is shorter than it is further east and north in Svalbard were observed to be in good condition. A “climate winner” in Svalbard is the harbour seal (*Phoca vitulina*), with an increasing population that is spreading along the coast^84^. Polar bears have been observed to successfully take harbour seals in Svalbard on several occasions in later years (pers. comm. J. Mosbacher, B Moe, D. Wojtanowicz).

Although the number of polar bears in the Barents Sea may still below carrying capacity, the population has been increasing after excessive hunting until 50 years ago. ^14,16^. A density below carrying capacity could contribute to the good body condition despite the significant reduction in sea ice habitat in the area. If this is the case, we could see a sudden decline in condition when the population reaches what likely is a declining carrying capacity.

Our bears showed a high level of resistance to environmental changes induced by a warmer climate and sea ice habitat loss. Still, the evidence is clear that the loss of sea ice has had negative effects on several other polar bear populations across the Arctic^9,61,85^. Any population will respond differently based on the variation in both intrinsic and extrinsic factors, and care needs to be taken when predicting how bears will be affected by climate change in the future. Our ability to better predict how polar bears may cope with a continued warmer climate demands continued research across more populations experiencing variable environments. Populations of polar bears occupy areas with highly diverse habitats and prey from Hudson Bay in the south to the high Arctic^69,86^. In some of these areas, reductions in sea ice may lead to transient periods where bears will do better in future, before continued loss of sea ice later may lead to a population decline^61,87^. The Barents Sea population, which has experienced the fastest loss of sea ice among all the polar bear populations^12^, is also predicted to have the most significant loss in future decades^88^. Besides local bears being forced on land and depending more on terrestrial food^24,61^, pelagic bears experienced longer distances between hunting grounds at the ice edge and denning and mating areas in Svalbard and Franz Josef Land^20^, with energetic demanding long swimming trips needed to travel between these areas^19,89^. For the pelagic bears, the fact that sea ice still is located over a large area of shallow water in the BS, in the area most used by the polar bears^13,15^, support findings by Rode et al.^90,91^ where bears having a large accessible shelf in the Chukchi Sea still do fine while bears having a much reduced area of sea ice over a shallow shelf in the Southern Beaufort Sea do worse. However, the still good condition among highly local Svalbard bears can only be explained by local factors.

Monitoring in the future will allow insight into how the two different ecotypes fare in a rapidly warming part of the high Arctic. The results from this paper, which show that polar bears have not yet experienced a decline in body condition in an area where sea ice habitat loss has occurred faster than anywhere else they live, illustrate how important it is not to extrapolate findings from one region to another. Given projections for continued warming, the clear negative effects of sea ice habitat loss on polar bears in other areas, and the fact that polar bears are not found anywhere that does not provide access to sea ice and their prey, bears in the BS area are likely to be negatively affected in the near future. This underscores the need for ongoing monitoring of both the bears and their ecosystem.

## Supplementary Information

Below is the link to the electronic supplementary material.

Supplementary Material 1

## Data Availability

The data used in the models are available at: [https://zenodo.org/records/15775231](https:/zenodo.org/records/15775231) . Also corresponding author can be contacted for access to the data and R-scripts.
