# Supplementary material for: Body condition among Svalbard Polar bears Ursus maritimus during a period of rapid loss of sea ice
Source: Sci Rep. 2026 Jan 29;16:2182. doi: 10.1038/s41598-025-33227-9 (PMC12855275; doi:10.1038/s41598-025-33227-9)
Supplement: Supplementary file 1 — Supplementary Material 1 [file 41598_2025_33227_MOESM1_ESM.pdf]

Supplementary Information

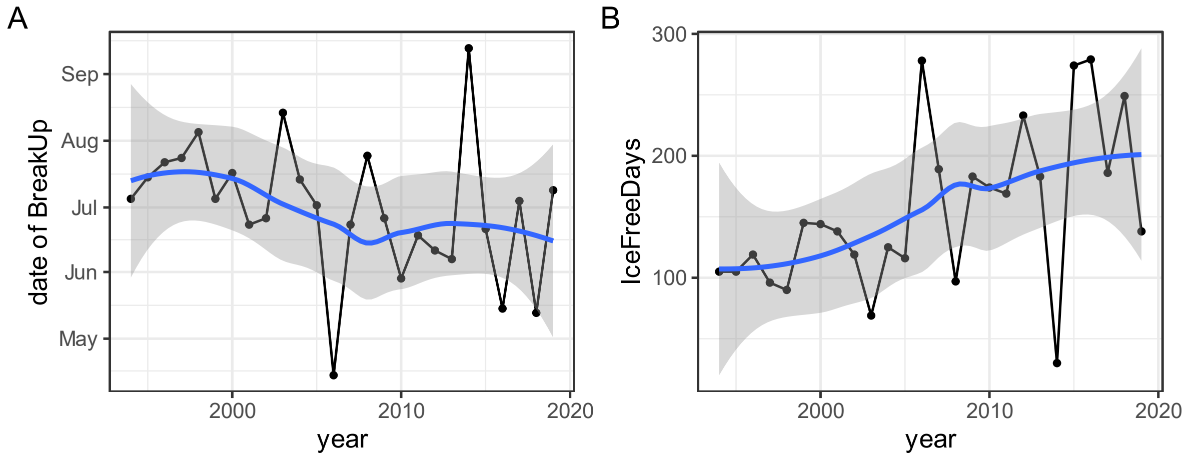

Supplementary Figure 1. The changes in sea ice metrics during the study period. Data includes 1994 (the year before the bear capture data), as several of the models on BCI (Body Condition Index) in year t includes either sea ice break up (BreakUp) or number of ice free days (IceFreeDays) in year t-1.

The trend line is drawn using loess smoothing function with the 95% CIs (dark grey envelopes).

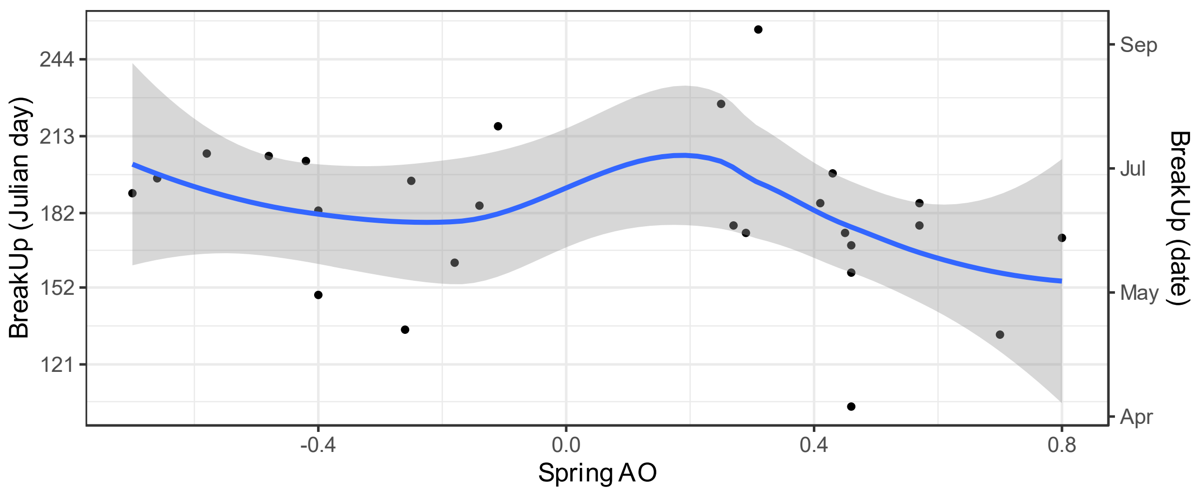

Supplementary Figure 2. the relationship between spring AO and day (Julian) of sea ice break up the same year.

Supplementary Table 1. Statistical values for model M10, the top selected model explaining the male body condition index (BCI).

	mean	sd	0.025q	0.5q	0.975q	mode
intercept	0.03	0.38	-0.76	0.02	0.82	0.02
Precision Gauss. Obs.	6.06	0.63	4.9	6.04	7.39	5.99
Precision Year	8.85	5.89	2.22	7.38	24.25	5.1
Precision Age	53.46	46.9	9.15	40.18	177.89	22.78
Precision DayM	1393.91	4761.86	25.82	402.54	8982.99	57.14
Range for w	434.23	356.09	81.81	335.74	1378.02	200.75
Stdev for w	0.21	0.08	0.09	0.2	0.41	0.17
Precision fBearID	10.11	2.24	6.53	9.83	15.31	9.23

DIC	718.7
DIC, Saturated	700.4
Eff. Numb. Param.	174.3
WAIC	726.1
Eff. Numb. Param.	146.5
Marg. Log-Likel.	-618.1

Supplementary Table 2. Statistical values for model M9a, the second ranked alternative model explaining the male body condition index (BCI).

	mean	sd	0.025q	0.5q	0.975q	mode
intercept	0.02	0.19	-0.39	0.01	0.44	0.01
Precision Gauss. Obs.	5.93	0.64	4.76	5.9	7.27	5.86
Precision springAO	74.96	131.55	4.52	37.65	382	11.47
Precision winterAO	530.33	1396.86	17.81	195.37	3170	42.73
Precision BreakUp	7.24	5.59	1.55	5.72	22.1	3.63
Precision Age	51.02	41.95	8.89	39.47	162	22.78
Precision DayM	2286.69	9021.78	31.58	573.76	15200	65.79
Range for w	279.83	272.52	38.98	200.43	1000	101.2
Stdev for w	0.19	0.07	0.09	0.18	0.35	0.16
Precision fBearID	11.01	2.64	6.82	10.67	17.2	9.98

DIC	729.7
DIC, Saturated	700.3
Eff. Numb. Param.	173.7
WAIC	737.5
Eff. Numb. Param.	146.7
Marg. Log-Likel.	-753

Supplementary Table 3. Statistical values for model F9a, the top selected model explaining the female BCI.

	mean	sd	0.025q	0.5q	0.975q	mode
intercept (with cubs)	-1.23	0.07	-1.37	-1.23	-1.1	-1.23
lone	0.35	0.05	0.26	0.35	0.44	0.35
with yearlings	0.15	0.07	0.02	0.15	0.28	0.15
Precision Gauss. Obs.	5.13	0.47	4.25	5.11	6.09	5.1
Precision springAO	115.94	227.86	4.57	52.59	625.78	11.26
Precision winterAO	261.69	534.97	12.53	117.56	1427.37	31.17
Precision BreakUp	17.24	19.8	2.4	11.39	68.66	5.69
Precision Age	1278.87	4770.27	11.67	321.56	8474.11	18.86
Precision DayM	699.19	1420.51	28.82	311.62	3821.02	71.24
Range for w	77.3	43.5	24.94	67.14	190.02	51.11
Stdev for w	0.17	0.04	0.1	0.17	0.27	0.16
Precision fBearID	25.13	9.66	12.11	23.24	49.41	19.81

DIC	957.2
DIC, Saturated	819.6
Eff. Numb. Param.	153.9
WAIC	963.4
Eff. Numb. Param.	136.1
Marg. Log-Likel.	-855.4

Supplementary Table 4. Statistical values for model F10, alternative model explaining the female BCI.

	mean	sd	0.025q	0.5q	0.975q	mode
intercept (with cubs)	-1.24	0.07	-1.38	-1.24	-1.09	-1.24
lone	0.37	0.05	0.28	0.37	0.46	0.37
with yearlings	0.16	0.07	0.03	0.16	0.29	0.16
Precision Gauss. Obs.	4.96	0.44	4.13	4.94	5.86	4.93
Precision Year	51.39	69.5	4.77	30.73	227	12.19
Precision Age	2274.83	10200	22.66	496.59	15400	43.29
Precision DayM	895.96	2230	28.86	341.57	5290	69.09
Range for w	81.77	41.8	29.88	72.38	189	57.24
Stdev for w	0.19	0.04	0.12	0.19	0.28	0.18
Precision fBearID	25.54	10	12.29	23.51	50.9	19.84

DIC	973.5
DIC, Saturated	814.8
Eff. Numb. Param.	149.5
WAIC	978.4
Eff. Numb. Param.	131.9
Marg. Log-Likel.	-728.6

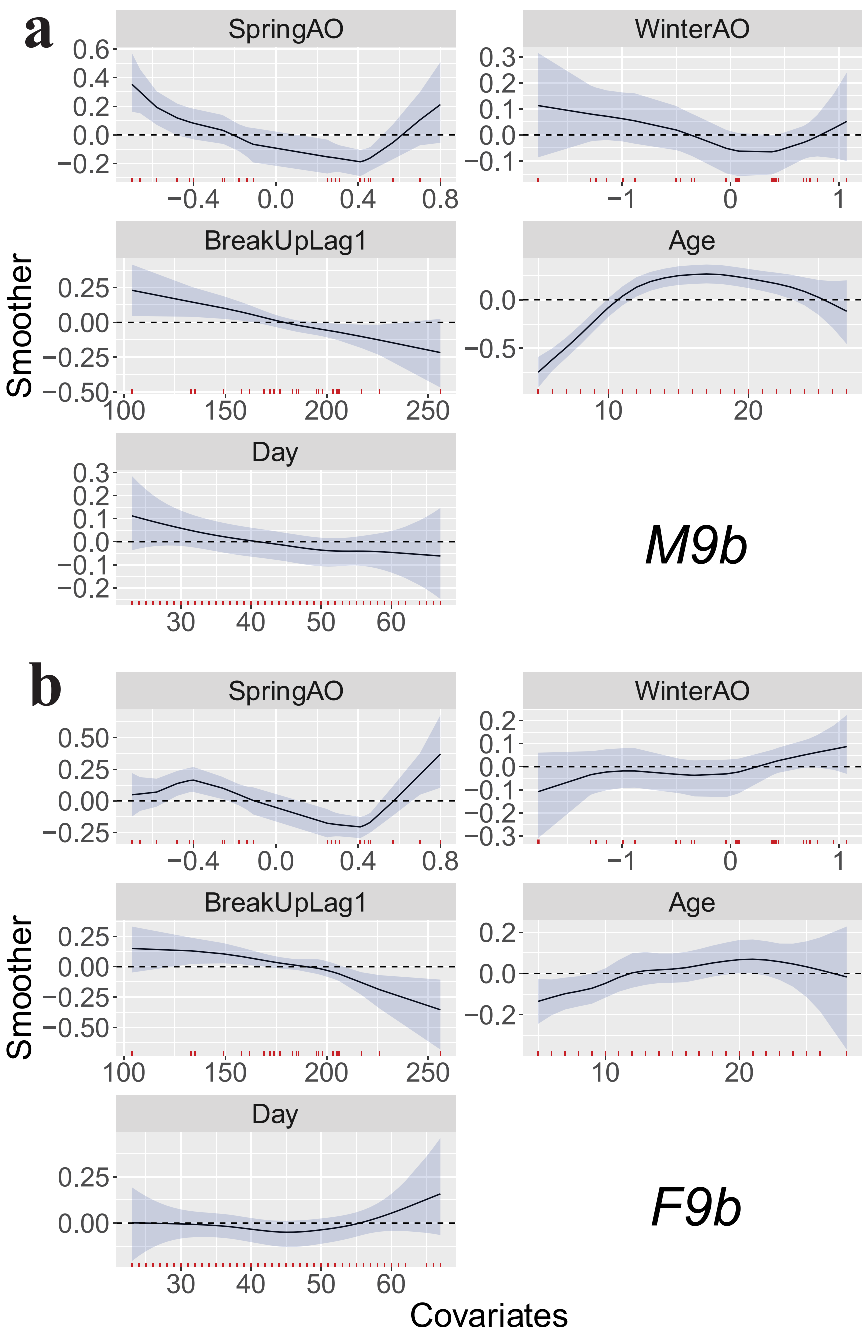

Supplementary Figure 3. Smoothers for the partial effects of the explanatory variables for (a) model M9b (males) and (b) model F9b (females).

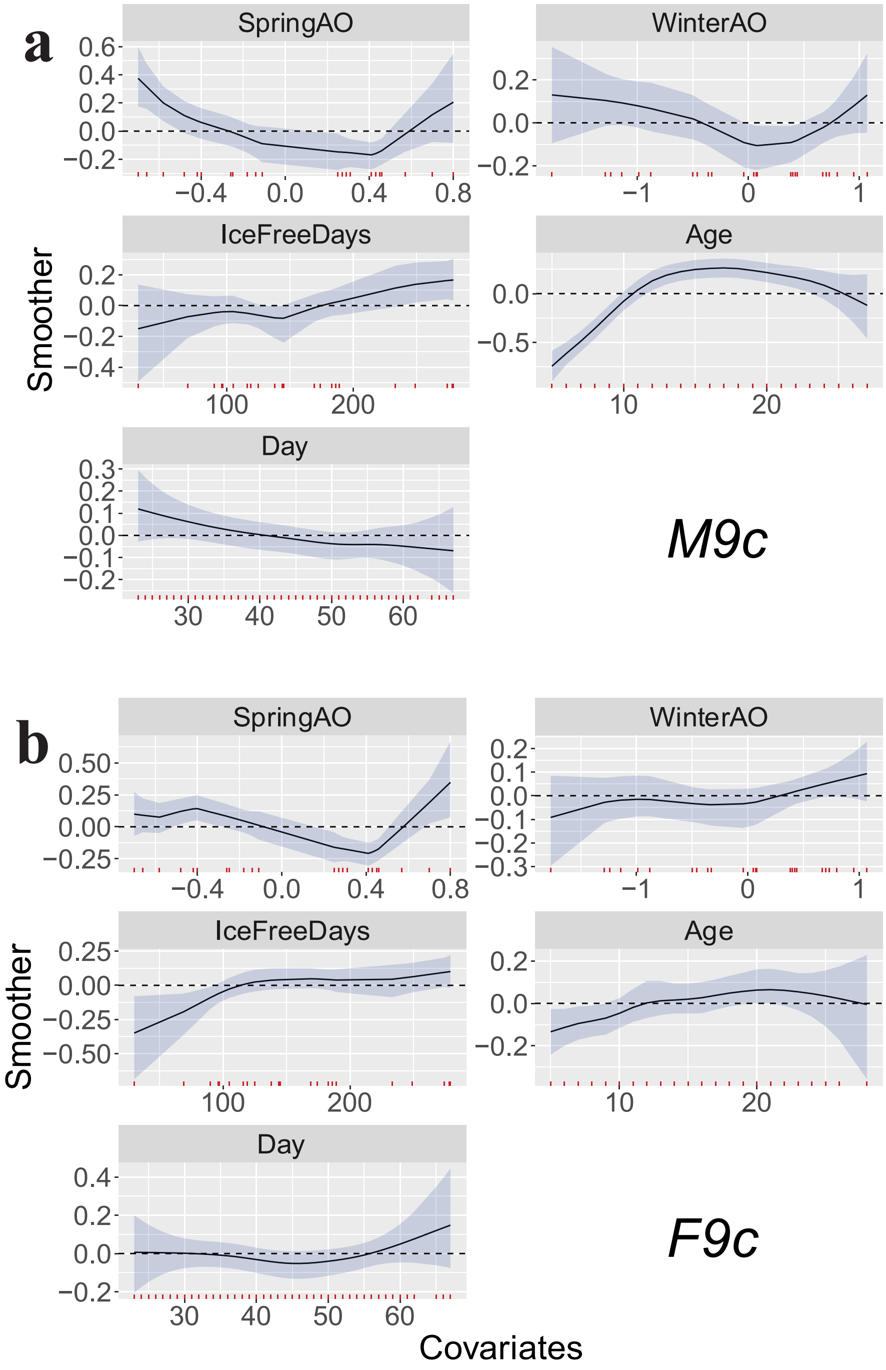

Supplementary Figure 4. Smoothers for the partial effects of the explanatory variables for (a) model M9c (males) and (b) model F9c (females).

Sensitivity Analysis of Mesh Resolution and Prior for the Spatial Range

A sensitivity analysis was conducted to assess the robustness of the spatial model to changes in mesh resolution and prior specification for the spatial range parameter. Six meshes of increasing coarseness (approximately 15,894 to 704 nodes) and five Penalised Complexity (PC) priors for the range parameter (defined by $P(\mathrm{Range} < x) = 0.05$, with $x = 30{,}35{,}40{,}45{,}50$) were combined, resulting in 30 unique SPDE models. Each model was fitted using **INLA** with the same fixed effects structure and random effects specification.

Model performance was evaluated using **DIC** and **R^2**, and spatial structure was compared via posterior mean range estimates and visual inspection of the spatial random fields.

Results summary:

- **Model fit:** DIC (for heatmap, see Fig. 5) and R^2 (for heatmap, see Fig. 6) values showed only minimal variation across all models, indicating that the results are robust to reasonable changes in mesh resolution and prior specification.
- **Posterior range:** Posterior mean values of the range varied slightly between priors, as expected, but remained within a consistent range.
- **Spatial random fields:** The spatial random fields were visually indistinguishable across models, confirming that spatial patterns were stable (see figure 7).
- **Mesh and prior interaction:** Only the combination of a **very coarse mesh** with an **unrealistically restrictive prior** produced noticeably different spatial dependence.

Conclusion:
The model is robust to the choice of mesh resolution and range prior within reasonable bounds. Only overly coarse meshes combined with overly restrictive range priors meaningfully affect the spatial dependence estimates.

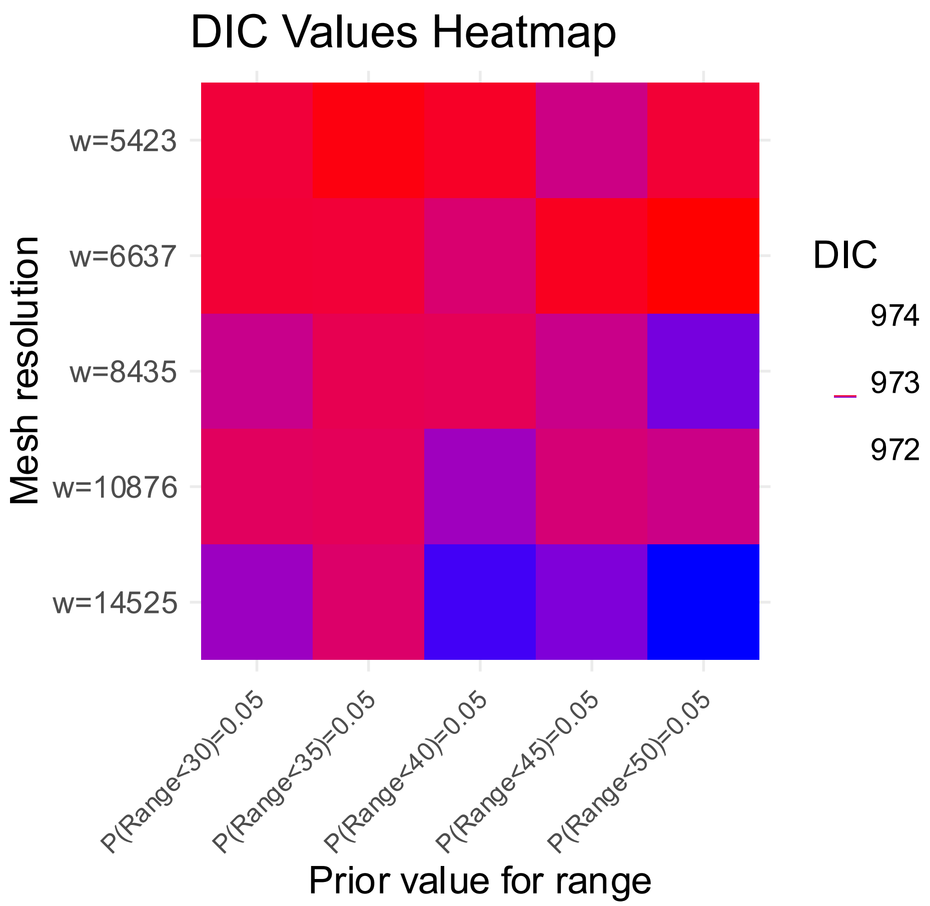

Supplementary Figure 5. Heatmap showing how the DIC varies with mesh resolution and the prior value for the range.

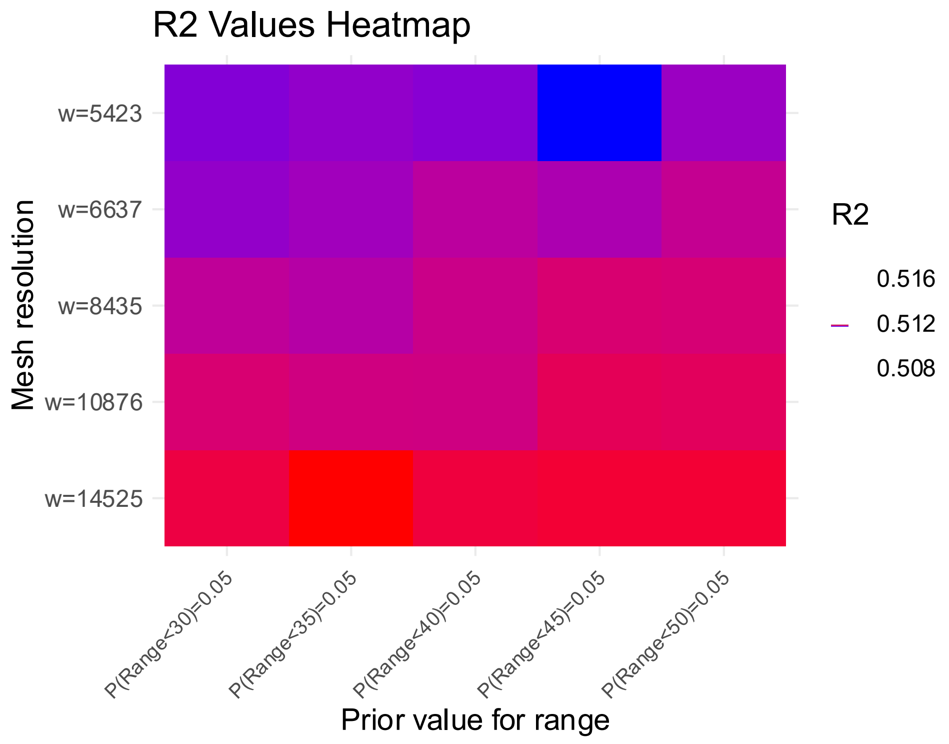

Supplementary Figure 6. Heatmap showing how the R^2 varies with mesh resolution and the prior value for the range.

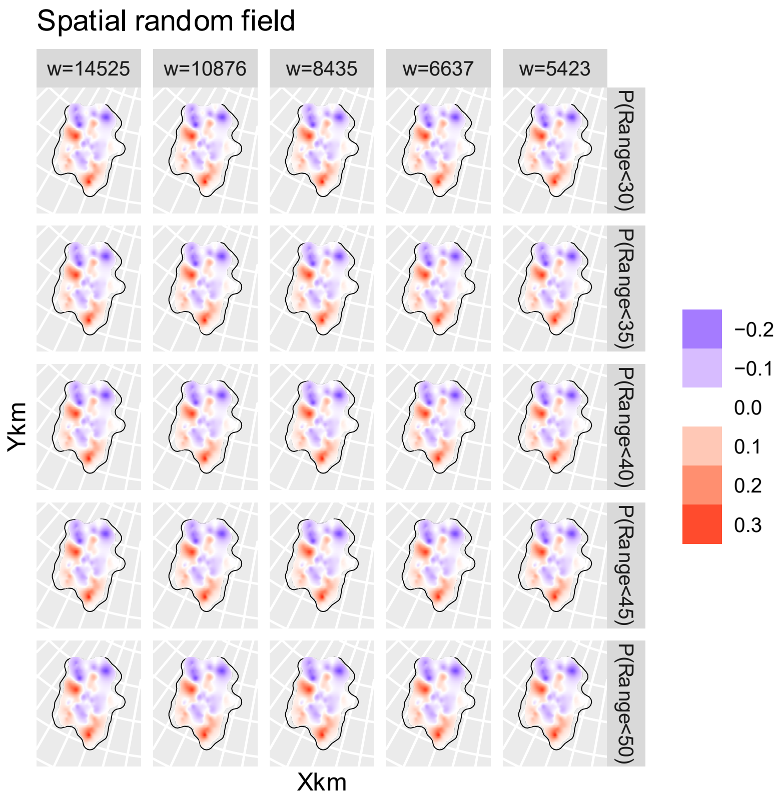

Supplementary Figure 7. Spatial random fields obtained using different mesh resolutions and priors for the range.
